# SIRT1 inhibits EV71 genome replication and RNA translation by interfering with the viral polymerase and 5′UTR RNA

**DOI:** 10.1242/jcs.193698

**Published:** 2016-12-15

**Authors:** Yang Han, Lvyin Wang, Jin Cui, Yu Song, Zhen Luo, Junbo Chen, Ying Xiong, Qi Zhang, Fang Liu, Wenzhe Ho, Yingle Liu, Kailang Wu, Jianguo Wu

**Affiliations:** State Key Laboratory of Virology and College of Life Sciences, Wuhan University, Wuhan 430072, China

**Keywords:** Enterovirus 71, HDAC, IRES, SIRT1, Viral infection, Replication, Transcription, Translation

## Abstract

Enterovirus 71 (EV71) possesses a single-stranded positive RNA genome that contains a single open reading frame (ORF) flanked by a 5′ untranslated region (5′UTR) and a polyadenylated 3′UTR. Here, we demonstrated that EV71 activates the production of silent mating type information regulation 2 homolog 1 (SIRT1), a histone deacetylase (HDAC). EV71 further stimulates SIRT1 sumoylation and deacetylase activity, and enhances SIRT1 translocation from the nucleus to the cytoplasm. More interestingly, activated SIRT1 subsequently binds with the EV71 3D^pol^ protein (a viral RNA-dependent RNA polymerase, RdRp) to repress the acetylation and RdRp activity of 3D^pol^, resulting in the attenuation of viral genome replication. Moreover, SIRT1 interacts with the cloverleaf structure of the EV71 RNA 5′UTR to inhibit viral RNA transcription, and binds to the internal ribosome entry site (IRES) of the EV71 5′UTR to attenuate viral RNA translation. Thus, EV71 stimulates SIRT1 production and activity, which in turn represses EV71 genome replication by inhibiting viral polymerase, and attenuates EV71 RNA transcription and translation by interfering with viral RNA. These results uncover a new function of SIRT1 and reveal a new mechanism underlying the regulation of EV71 replication.

## Abstract (precis)

**Summary:** EV71 infection is a hazard to children. This study reveals a new mechanism underlying EV71 replication and suggest that SIRT1 could be an agent for the treatment of the viral infection.

## INTRODUCTION

Enterovirus 71 (EV71) infection causes herpangina, hand-foot-mouth disease (HFMD), meningoencephalitis, aseptic meningitis, encephalitis, acute flaccid paralysis and possibly fatal encephalitis (Weng et al., 2010). EV71 belongs to the *Enterovirus* genus of the Picornaviridae family, and is a non-enveloped virus with a positive single-stranded RNA genome that contains a single open reading frame (ORF) flanked by a 5′ untranslated region (5′UTR) and a polyadenylated 3′UTR (McMinn, 2002). The viral genome encodes a 250-kDa polyprotein that is processed into one structural (P1) and two nonstructural (P2 and P3) regions, which are further cleaved into precursors and mature proteins (VP1 to VP4, 2A to 2C, and 3A to 3D) (Solomon et al., 2010). Viral polyprotein processing is mediated by two proteases (2A^pro^ and 3C^pro^) (Wu et al., 2010). Among the mature proteins, EV71 3D^pol^ protein (3D^pol^) acts as a viral RNA-dependent RNA polymerase (RdRp) and plays a major role in viral genome synthesis (Richards et al., 2006; Rueckert, 1996). The EV71 5′UTR RNA contains a cloverleaf structure involved in viral RNA transcription and an internal ribosome entry site (IRES) that mediates translation initiation (Rohll et al., 1994). The cloverleaf structure (stem-loop I) is essential for negative-strand synthesis, which requires a membrane-associated replication complex of viral RNA template along with viral and cellular proteins (Barton et al., 2001; Lyons et al., 2001). The IRES structure (stem-loops II–VI) is required for viral RNA translation initiation through a cap-independent mechanism (Thompson and Sarnow, 2003).

Silent mating type information regulation 2 homolog 1 (SIRT1) is a member of the sirtuin family, which contains seven proteins (SIRT1–SIRT7) that are class III NAD^+^-dependent histone deacetylases (HDACs) (Bannister and Kouzarides, 2011). The genes encoding this group are highly conserved among the genomes of organisms ranging from archaebacteria to eukaryotes (Blander and Guarente, 2004; Frye, 2000; North and Verdin, 2004; Sauve et al., 2006). SIRT1 deacetylates a wide range of substrates with roles in cellular processes ranging from energy metabolism to cell survival (Guarente, 2007). SIRT1 also regulates human immunodeficiency virus 1 (HIV-1) transcription through Tat deacetylation (Pagans et al., 2005), and hepatitis B virus (HBV) replication by targeting the transcription factor AP-1 (Ren et al., 2014).

In this study, we revealed a new mechanism underlying the regulation of EV71 replication that is mediated by SIRT1. We demonstrated that EV71 infection activates SIRT1 production, sumoylation and deacetylase activity, and enhances SIRT1 translocation from the nucleus to the cytoplasm. Subsequently, EV71-activated SIRT1 binds with the viral 3D^pol^ protein and attenuates the acetylation and RdRp activity of 3D^pol^, resulting in the repression of viral genome replication. Moreover, SIRT1 interacts with the cloverleaf structure of EV71 5′UTR to repress viral RNA transcription, and binds to the IRES of EV71 5′UTR to attenuate viral RNA translation. These results reveal a new mechanism underlying the regulation of EV71 replication mediated by SIRT1, and suggested that SIRT1 might function as a potential agent for the prevention and treatment of the diseases caused by EV71 infection.

## RESULTS

### EV71 facilitates SIRT1 production and enhances SIRT1 translocation from the nucleus to the cytoplasm

**Fig. 1.**
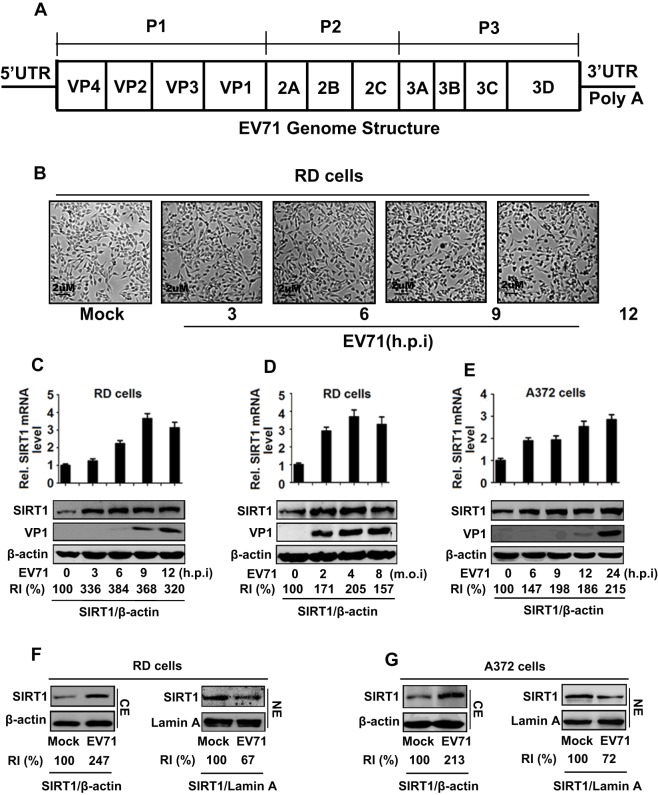
**EV71 enhances SIRT1 expression and translocation.** (A) A diagram of the EV71 genome structure. EV71 genome contains a single ORF flanked by a 5′UTR and a 3′UTR. The ORF encodes a 250-kDa polyprotein that is processed into P1, P2 and P3 regions, which are further cleaved into mature proteins (VP1 to VP4, 2A to 2C, and 3A to 3D^pol^). (B) RD cells were infected with EV71 at a multiplicity of infection (MOI) of 5 for different times. Photographs of infected cells were taken using a digital camera (at 100× magnification). (C–E) RD cells were infected with EV71 at an MOI of 5 for different times (C). RD cells were infected with EV71 for 12 h at different MOI (D). SK-N-SH A372 cells were infected with EV71 at an MOI of 5 for different times (E). The relative amount of SIRT1 and VP1 mRNAs were determined by qRT-PCR (upper panels). SIRT1 and VP1 proteins were detected by western blot analyses using corresponding antibodies (lower panels). (F,G) RD cells (F) and SK-N-SH A372 cells (G) were infected with EV71 at an MOI of 5 for 6 h. Cytoplasm extracts (CE) and nuclear extracts (NE) were prepared. SIRT1, β-actin and lamin A were detected by western blot analyses using corresponding antibodies. Each experiment was performed in triplicate wells and repeated at least three times. The intensity of western blot bands signals were quantified with Image J. RI, relative intensity.

The EV71 genome is a positive single-stranded RNA of ∼7.5 kb in size flanked by the 5′UTR and 3′UTR (Fig. 1A). The viral single ORF encodes a polyprotein that is subdivided into three regions, P1, P2 and P3. P1 encodes four structural viral proteins (VP1–VP4), P2 encodes three non-structural proteins (2A–2C), and P3 encodes four non-structural proteins (3A–3D). We initially investigated the effects of EV71 on SIRT1 expression. EV71 caused cytopathogenic effects on infected human rhabdomyosarcoma (RD) cells in a time-dependent manner (Fig. 1B), indicating that the infection was effective. SIRT1 mRNA and SIRT1 protein were upregulated by EV71 starting at 3 h post-infection (hpi) (Fig. 1C) (*P*<0.05) and further increased by EV71 infection at an multiplicity of infection (MOI) of 2 in RD cells (Fig. 1D) (*P*<0.05). SIRT1 mRNA and protein were also upregulated by EV71 in human neurosarcoma cells (SK-N-SH/A372) (Fig. 1E) (*P*<0.05), revealing that EV71 facilitates SIRT1 expression at transcriptional and translational levels. The effect of EV71 on the translocation of SIRT1 was then evaluated. In EV71-infected RD cells, SIRT1 protein was increased in the cytoplasm (Fig. 1F, left), but decreased in the nucleus (Fig. 1F, right) (*P*<0.05); in infected SK-N-SH cells, SIRT1 protein was also increased in the cytoplasm (Fig. 1G, left), but not in the nucleus (Fig. 1G, right) (*P*<0.05). These results suggest that EV71 attenuates the SIRT1 nuclear translocation in infected cells.

### SIRT1 is sumoylated by Sumo1 and EV71 facilitates SIRT1 sumoylation

**Fig. 2.**
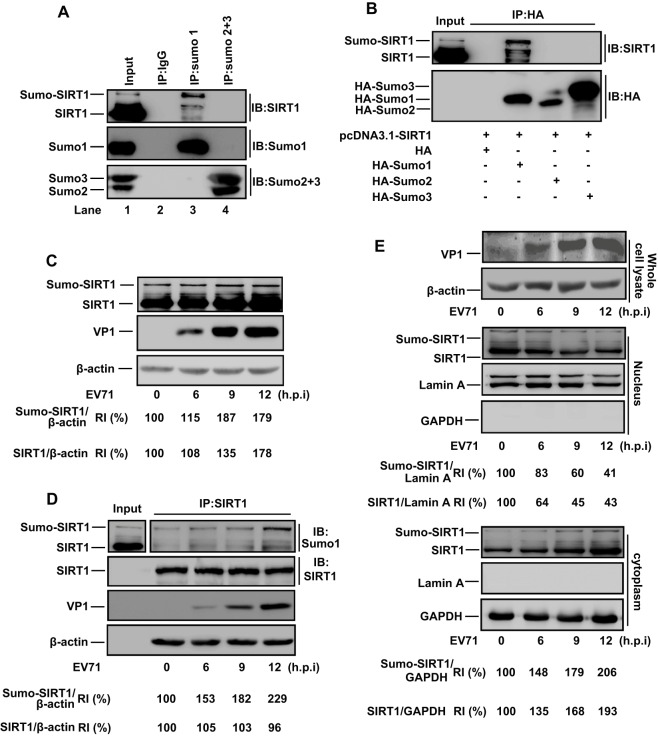
**SIRT1 is sumoylated with Sumo1 and EV71 facilitates SIRT1 sumoylation.** (A) RD cells were harvested and incubated in RIPA buffer for 20 min. Cell lysates were centrifuged at 20,000 ***g*** for 15 min to remove cellular debris. IgG, anti-Sumo1 antibody, anti-Sumo2+3 antibody (i.e. an antibody that could recognize both Sumo-2 and Sumo-3) or protein G (IP) was added to supernatants for immunoprecipitation (IP). Sumoylated SIRT1 (Sumo-SIRT1) and SIRT1 were detected with an anti-SIRT1 antibody (IB). (B) RD cells were co-transfected with pcDNA3.1-SIRT1 and plasmid expressing HA–Sumo1, HA–Sumo2 or HA–Sumo3. Cell lysates were prepared and immunoprecipitated with anti-HA antibody. Precipitated sumoylated SIRT1 and SIRT1 were detected with an anti-SIRT1 antibody. (C) RD cells were infected with EV71 for different times. Infected cell lysates were prepared for western blotting using anti-SIRT1 or anti-VP1 antibodies. (D) RD cells were infected with EV71 for different times. Cell lysates were prepared and immunoprecipitated with anti-SIRT1 antibody, and Sumo–SIRT1 was detected with anti-Sumo1 antibody. (E) RD cells were infected with EV71 for different times. Whole-cell lysates (WCL) were prepared for detecting EV71 replication (top). Nuclear extracts (NE) (middle) and cytoplasm extracts (CE) (bottom) were prepared. The levels of Sumo–SIRT1, SIRT1, lamin A and GAPDH were determined by western blot analyses with the corresponding antibodies. Each treatment was repeated three or more times. The intensity of the western blot signals was quantified with Image J. RI, relative intensity.

Sumoylation of SIRT1 stimulates its deacetylase activity and cellular response to genotoxic stress (Yang et al., 2007). Thus, the effect of EV71 on SIRT1 sumoylation was evaluated. SIRT1 was sumoylated with Sumo1, but not with Sumo2 or Sumo3, in RD cells (Fig. 2A). RD cells were co-transfected with pcDNA3.1-SIRT1 and plasmids expressing HA alone, HA–Sumo1, HA–Sumo2 or HA–Sumo3. Cell lysates were incubated with anti-HA antibody and protein-G–agarose. The results confirmed that SIRT1 was sumoylated with HA–Sumo1, but not with HA–Sumo2 or HA–Sumo3 (Fig. 2B).

The effect of EV71 on SIRT1 sumoylation was evaluated in RD cells infected with EV71 for different times (as for Fig. 1C,D). We showed that SIRT1 and sumoylated SIRT1 proteins were upregulated by EV71 in RD cells (Fig. 2C,D) (*P*<0.05). The role of EV71 in endogenous SIRT1 sumoylation was also determined in RD cells infected with EV71 for different times, as indicated (Fig. 2E). Whole-cell lysates (WCL), nuclear extracts and cytoplasmic extracts were prepared and incubated with corresponding antibodies or protein G. We found that EV71 VP1 could be detected in WCL (Fig. 2E, top), and that SIRT1 and sumoylated SIRT1 proteins were decreased in nuclear extracts (Fig. 2E, middle) but increased in cytoplasmic extracts compared to in uninfected cells (Fig. 2E, bottom). Taken together, these results show that SIRT1 is modified by Sumo1, and EV71 facilitates SIRT1 sumoylation and translocation of sumoylated SIRT1 from the nucleus to the cytoplasm.

### SIRT1 inhibits EV71 replication in the cytoplasm of infected cells

**Fig. 3.**
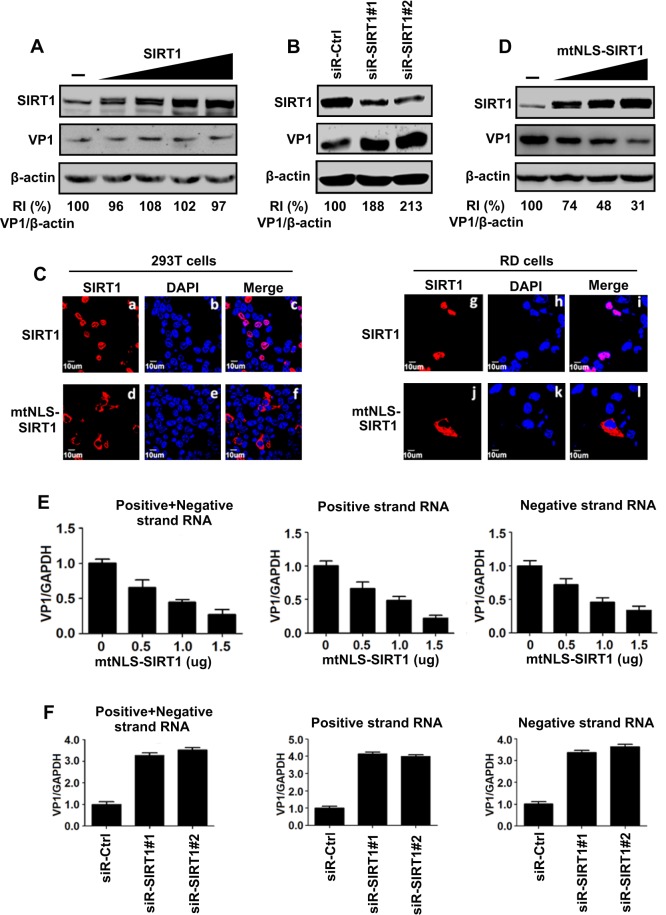
**SIRT1 inhibits EV71 replication in cytoplasm of infected cells.** (A) RD cells were transfected with pcDNA3.1(+)-SIRT1 at 0, 0.5, 1, 1.5 and 2 µg for 24 h and infected with EV71 at an MOI of 5 for 12 h. Cell lysates were prepared. SIRT1, EV71 VP1 and β-actin were detected by western blot analyses with corresponding antibodies. (B) RD cells were transfected with siR-Ctrl, siR-SIRT1#1 or siR-SIRT1#2, for 24 h, and infected with EV71 at an MOI of 5 for 12 h. SIRT1, VP1 and β-actin in cell lysates were detected by western blot analysis. (C) 293T and RD cells were transfected with plasmids expressing wild-type SIRT1 (WT-SIRT1) and SIRT1 with a mutant NLS (mtNLS-SIRT1). The cells were fixed, permeabilized and immunostained with antibody against SIRT1 (a,d,g,j), with Cy3-conjugated goat anti-rabbit-IgG used as a secondary antibody. The nucleus was stained with DAPI (b,e,h,k). The immunofluorescence results were analyzed by confocal laser-scanning microscopy. (D) RD cells were transfected with plasmids expressing mtNLS-SIRT1 at 0, 0.5, 1 and 1.5 µg for 24 h, and infected with EV71 infected at an MOI of 5 for 12 h. VP1 and β-actin in cell lysates were detected by western blot analysis. (E) RD cells were transfected with plasmids expressing mtNLS-SIRT1 at different concentrations for 24 h, and infected with EV71 at an MOI of 5 for 12 h or (F) RD cells were transfected with siR-Ctrl, siR-SIRT1#1 or siR-SIRT1#2 at 5 µM for 24 h, and infected with EV71 at an MOI of 5 for 12 h. Cells were harvested and total mRNA was isolated by using Trizol. The levels of GAPDH mRNA, EV71 VP1 double-strand RNA, positive-strand RNA and negative-strand RNA were determined by qRT-PCR. Ratios of positive-strand RNA to GAPDH mRNA, positive-strand RNA to GAPDH mRNA and negative-strand RNA to GAPDH mRNA were calculated. Results are mean±s.e.m. (*n*=5). The intensity of western blot signals was quantified with Image J. RI, relative intensity.

As SIRT1 expression and activity were regulated by EV71, we wanted to determine whether SIRT1 plays a role in EV71 infection. The level of EV71 VP1 protein was relatively unaffected by SIRT1 in infected RD cells (Fig. 3A), indicating that overexpression of SIRT1 has no effect on EV71 protein production. However, the level of EV71 VP1 protein was significantly higher (*P*<0.05) in the presence of short interfering RNAs (siRNAs) against SIRT1 (denoted siR-SIRT1#1 or siR-SIRT1#2; Fig. 3B), suggesting that knockdown of SIRT1 upregulates VP1 production. We speculated that this discrepancy might be due to the different subcellular localizations of EV71 and SIRT1, because SIRT1 is mainly localized in the nucleus, whereas EV71 replication occurs in cytoplasm, and EV71 attenuates the SIRT1 nuclear translocation in infected cells.

To investigate this phenomenon, we evaluated the subcellular localizations of wild-type SIRT1 and mtNLS-SIRT1 [an SIRT1 mutant in which the nuclear localization signal (NLS) is mutated]. SIRT1 was detected only in the nucleus of 293T (Fig. 3Ca,c) and RD cells (Fig. 3Cg,i), whereas mtNLS-SIRT1 was detected mainly in the cytoplasm of 293T (Fig. 3Cd,f) and RD cells (Fig. 3Cj,l), indicating that the nuclear localization of mtNLS-SIRT1 is abolished upon the mutation of the NLS. The effect of mtNLS-SIRT1 on EV71 replication was determined in infected RD cells transfected with pcDNA3.1(+)-mtNLS-SIRT1. EV71 VP1 protein (Fig. 3D), double-strand RNA (dsRNA; Fig. 3E, left), positive-strand RNA (Fig. 3E, middle) and negative-strand RNA (Fig. 3E, right) were all significantly downregulated (*P*<0.05) upon expression of mtNLS-SIRT1, indicating that mtNLS-SIRT1 plays an inhibitory role in EV71 replication. In addition, EV71 VP1 dsRNA, positive-strand RNA and negative-strand RNA were all upregulated upon expression of siR-SIRT1#1 and siR-SIRT1#2 (Fig. 3F) (*P*<0.05), suggesting that knockdown of SIRT1 upregulates EV71 replication. Therefore, SIRT1 changes its location from the nucleus to the cytoplasm and plays an inhibitory role in EV71 replication.

### SIRT1 interacts with EV71 3D^pol^ both *in vivo* and *in vitro*

**Fig. 4.**
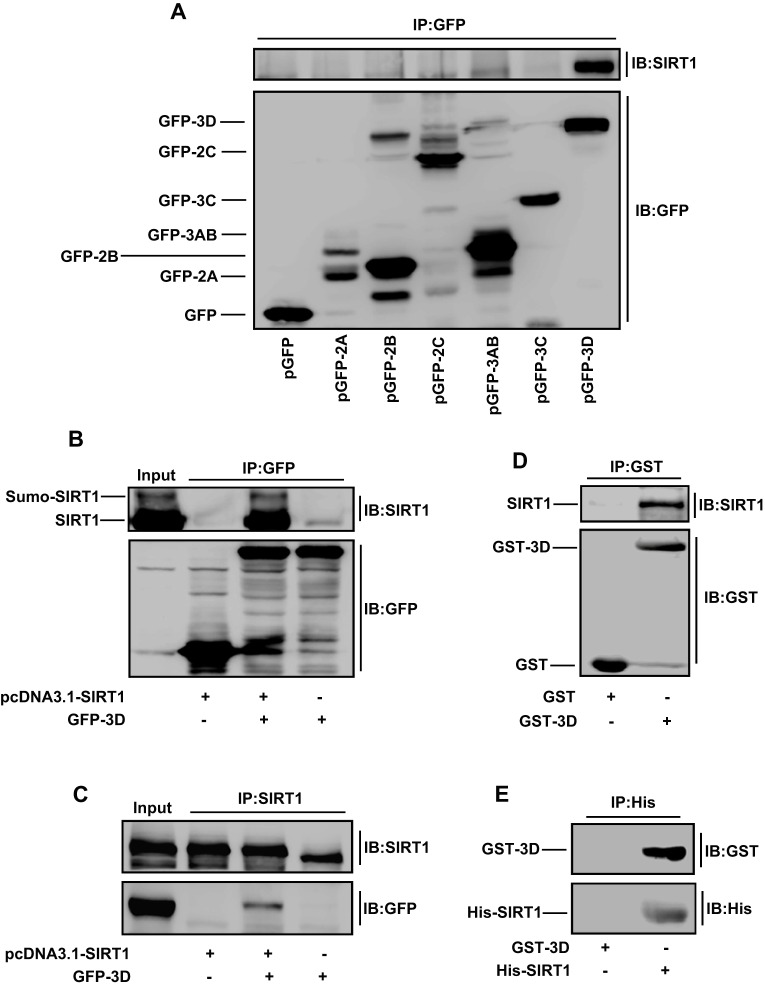
**SIRT1 interacts with EV71 3D^pol^ both *in vivo* and *in vitro*.** (A,B) 293T cells were co-transfected with pcDNA3.1(+)-SIRT1 and plasmids pGFP-C1, pGFP-2A, pGFP-2B, pGFP-3AB, pGFP-3C or pGFP-3D (A) or pGFP-3D (B). Cells extracts were prepared for co-immunoprecipitation (IP) using anti-GFP antibody and precipitated with protein G. Interactions between SIRT1 and viral proteins were detected by western blot analyses (IB) using anti-SIRT1 antibody or anti-GFP antibody. (C) 293T cells were co-transfected with pGFP-3D and pcDNA3.1(+)-SIRT1. Cell extracts were prepared for co-immunoprecipitation using anti-SIRT1 antibody and precipitated with protein G. The interaction between SIRT1 and GFP–3D^pol^ was detected by western blot analyses using anti-GFP antibody or anti-SIRT1 antibody. (D) 293T cells were transfected with pcDNA3.1(+)-SIRT1 for 48 h. Cell lysates were prepared, to which purified GST or GST–3D^pol^ were added, and were then purified by using a glutathione–Sepharose column and GST-binding buffer. Proteins were pulled down with anti-GST antibody, and interactions between SIRT1 and GST–3D^pol^ were detected by western blotting with anti-SIRT1 antibody or anti-GST antibody. (E) GST–3D^pol^ was incubated with His–SIRT1, and protein–protein pulldown assays were carried out with anti-His antibody. Interactions between His–SIRT1 and GST–3D^pol^ were determined by western blotting with anti-GST antibody or anti-His antibody.

Next, we investigated the mechanism by which SIRT1 represses EV71 replication. We speculated that SIRT1 might interact with non-structural proteins of EV71 to regulate their functions. To confirm this speculation, 293T cells were co-transfected with pcDNA3.1(+)-SIRT1 and plasmids expressing each of GFP-tagged EV71 non-structural proteins, 2A, 2B, 2C, 3AB, 3C and 3D^pol^. SIRT1 was detected in the co-immunoprecipitation with GFP–3D^pol^, but not with those of the other fusion proteins (Fig. 4A), indicating that SIRT1 only interacts with EV71 3D^pol^, an RNA-dependent RNA polymerase (RdRp) essential for viral genome replication.

The ability of SIRT1 to bind to 3D^pol^ was verified in 293T cells co-transfected with pGFP-3D^pol^ and pcDNA3.1(+)-SIRT1. SIRT1 was detected only upon immunoprecipitation of GFP–3D^pol^ (Fig. 4B), confirming that SIRT1 interacts with GFP–3D^pol^; sumoylated SIRT1 was also pulled down by GFP–3D^pol^, suggesting that sumoylated SIRT1 interacts with 3D^pol^ (Fig. 4B). Similarly, GFP–3D^pol^ was detected upon immunoprecipitation of SIRT1 (Fig. 4C), demonstrating that GFP–3D^pol^ interacts with SIRT1. The interaction between SIRT1 and 3D^pol^ was further explored by protein–protein pulldown assays. SIRT1 was detected upon pulldown of GST–3D^pol^ (Fig. 4D), suggesting that SIRT1 binds to 3D^pol^. Furthermore, recombinant GST–3D^pol^ was detected upon pulldown with recombinant His–SIRT1 (Fig. 4E), revealing that His–SIRT1 interacts with GST–3D^pol^. Taken together, these results show that SIRT1 binds to EV71 3D^pol^ both *in vivo* and *in vitro*.

### SIRT1 inhibits EV71 RNA replication through interacting with 3D^pol^ and repressing 3D^pol^ acetylation and RdRp activity

**Fig. 5.**
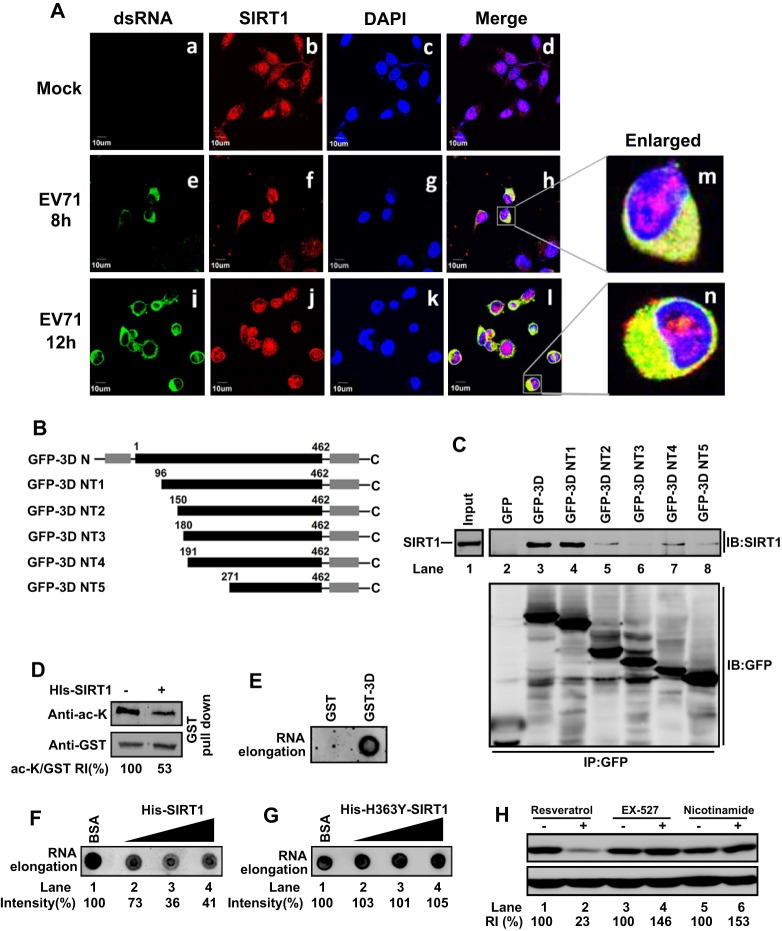
**SIRT1 inhibits EV71 genome RNA replication by interacting with 3D^pol^.** (A) RD cells were infected with EV71 or not (Mock) at an MOI of 5 for 8 h or 12 h, fixed, permeabilized, and immunostained with antibody against 3D^pol^ (a,e,i) or SIRT1 (b,f,j). Nuclei were stained by DAPI (c,g,k). FITC-conjugated donkey anti-mouse-IgG or Cy3-conjugated goat anti-rabbit-IgG was used as a secondary antibody. Immunofluorescence was detected by confocal laser-scanning microscopy. (B) Schematic diagram of the truncated EV71 3D^pol^ constructs used in this study, with amino acid numbers indicated. DNA fragments containing mutant EV71 3D^pol^ genes (3D-NT1, 3D-NT2, 3D-NT3, 3D-NT4 and 3D-NT5) were sub-cloned into plasmid peGFP-C1 to generate plasmids peGFP-C1-3D-NT1 to -NT5, respectively. N-terminal and C-terminal domains are indicated. (C) 293T cells were co-transfected with pcDNA3.1(+)-SIRT1 and peGFP-C1, peGFP-C1-3D^pol^ and peGFP-C1-3D-NT1–NT5 for 24 h. Cell extracts were prepared for co-immunoprecipitation (IP) assays using anti-GFP antibody and precipitated with protein G. Interactions between SIRT1 and EV71 proteins were determined by western blotting using anti-SIRT1 antibody or anti-GFP antibody. (D) Purified GST–3D^pol^ was incubated with p300 protein in acetylation assay buffer. Acetylated GST–3D^pol^ (0.02 µg) was incubated with His–SIRT1 (0.02 µg) in deacetylation assay buffer at 37°C for 30 min. The levels of acetylated (ac-K) GST–3D^pol^ and nonacetylated GST–3D^pol^ were determined by western blotting with anti-acetylated-lysine antibody or anti-GST antibody. (E–G) Purified GST or GST–3D^pol^ were incubated in acetylation assay buffer with p300 protein; then, acetylated GST and GST–3D^pol^ were incubated with His–SIRT1 in deacetylation assay buffer at 37°C for 30 min (E,F), or acetylated GST-3D^pol^ was incubated with His–H363Y-SIRT1 in deacetylation assay buffer for 30 min at 37°C (G). Mixtures were applied onto glutathione–Sepharose columns, gently rotated at 4°C for 3 h, incubated in RNA elongation assay buffer with DIG-UTP and then spotted onto Hybond-N membrane. Synthetic RNAs were detected using a Luminescent Image Analyzer. (H) RD cells were incubated with DMSO, resveratrol (an activator of SIRT1), EX-527 (an inhibitor of SIRT1) or nicotinamide (an inhibitor of SIRT1) for 6 h, and infected with EV71 at an MOI of 5 for 10 h. The levels of VP1 and β-actin were determined by western blot analysis. The intensity of the western blot signals was quantified with Image J. RI, relative intensity.

The subcellular distributions of SIRT1 and EV71 3D^pol^ in RD cells were analyzed by laser-scanning confocal microscopy. In mock-infected cells, 3D^pol^ was not detected (Fig. 5Aa), whereas endogenous SIRT1 was mainly localized in nuclei (Fig. 5Ab). In EV71-infected cells, at 8 h and 12 hpi, 3D^pol^ was detected in the cytoplasm (Fig. 5Ae,i), and a large proportion of SIRT1 was also distributed in the cytoplasm (Fig. 5Af,j), where it colocalized with 3D^pol^ (Fig. 5Ah,l, enlarged in Fig. 5Am,n), suggesting that SIRT1 changes its location from the nucleus to the cytoplasm upon interacting with 3D^pol^.

SIRT1 catalyzes an NAD^+^–nicotinamide exchange reaction that requires an acetylated lysine residue (Landry et al., 2000). 3D^pol^ contains an index finger domain (residues 1–68), a RING finger domain (150–179), a pinky finger domain (96–149 and 180–190), a middle finger domain (270–286), a palm domain (191–269, 287–381) and a thumb domain (382–462) (Wu et al., 2010). We constructed a series of mutant GFP–3D^pol^ proteins (3D^pol^ NT1 to NT5), in which the N-terminus of GFP–3D^pol^ was gradually truncated (Fig. 5B). In transfected 293T cells, SIRT1 was detected in the input (Fig. 5C, lane 1), and was not immunoprecipitated with GFP alone (as a negative control) (Fig. 5C, lane 2); however, it was immunoprecipitated by GFP–3D^pol^ (as a positive control) (Fig. 5C, lane 3) and GFP–3D^pol^ NT1 (Fig. 5C, lane 4), but was barely detected when GFP–3D^pol^ NT2 to NT5 (Fig. 5C, lanes 5–8) were expressed; this indicates that residues 96–149 of pinky finger domain of 3D^pol^ are required for the interaction with SIRT1. The pinky finger domain (residues 96–149 and residues 180–190) forms the front side of the fingers. The palm structure is the most conserved subdomain among RdRps and deletion of pinky finger domain might affect 3D^pol^ structure and activity (Gruez et al., 2008). Therefore, SIRT1 is colocalized with 3D^pol^ in the cytoplasm through interactions with pinky finger domain, which might lead to a repression of 3D^pol^ function.

Sumoylation of SIRT1 is required for its deacetylase activity, and as EV71 enhances SIRT1 sumoylation, we speculated that SIRT1 might affect 3D^pol^ acetylation to regulate its function. To confirm this speculation, recombinant GST–3D^pol^ was purified and incubated with p300 protein (a histone acetyltransferase) in acetylation assay buffer, and acetylated GST–3D^pol^ was incubated with recombinant His–SIRT1 in deacetylation assay buffer. GST–3D^pol^ acetylation was significantly reduced by His–SIRT1 (Fig. 5D) (*P*<0.05), suggesting that SIRT1 attenuates 3D^pol^ acetylation.

The effect of SIRT1-mediated deacetylation of 3D^pol^ on RdRp activity was determined by using an acetylation assay in combination with an RNA elongation assay. RNA synthesis was enhanced by GST–3D^pol^ and not by GST (Fig. 5E), indicating that GST–3D^pol^ has RdRp activity. The role of SIRT1 in regulation of 3D^pol^ RdRp activity was determined. RNA synthesis was stimulated by GST–3D^pol^ (Fig. 5F, lane 1), but significantly reduced (*P*<0.05) by SIRT1 (Fig. 5F, lanes 2–4), indicating that SIRT1 represses 3D^pol^ RdRp activity by inhibiting its acetylation. To verify specificity of SIRT1 function in inhibition of 3D^pol^ RdRp activity, we generated a mutant SIRT1 protein (His–H363Y-SIRT1). The level of DIG-UTP-labeled RNA synthesis stimulated by 3D^pol^ (Fig. 5G, lane 1) was not diminished under the influence of His–H363Y-SIRT1 (Fig. 5G, lanes 2–4), suggesting that H363Y-SIRT1 fails to inhibit 3D^pol^ activity. Therefore, SIRT1 inhibits EV71 replication by repressing 3D^pol^ RdRp activity.

Moreover, the effect of SIRT1 on EV71 replication was investigated in infected RD cells treated with SIRT1 activators (resveratrol) or inhibitors (EX527 and nicotinamide). EV71 VP1 was downregulated by resveratrol (Fig. 5H, lane 2 versus 1), but upregulated by EX527 (Fig. 5H, lane 4 versus 3) and nicotinamide (Fig. 5H, lane 6 versus 5), indicating that activation of SIRT1 downregulates VP1, whereas inhibition of SIRT1 upregulates VP1. As these compounds affect SIRT1 deacetylation activity, it is reasonable to suggest that SIRT1 inhibits EV71 replication by downregulating 3D^pol^ acetylation and RdRp activity, leading to inhibition of viral RNA synthesis.

### SIRT1 binds directly to EV71 5′UTR, but not 3′UTR

**Fig. 6.**
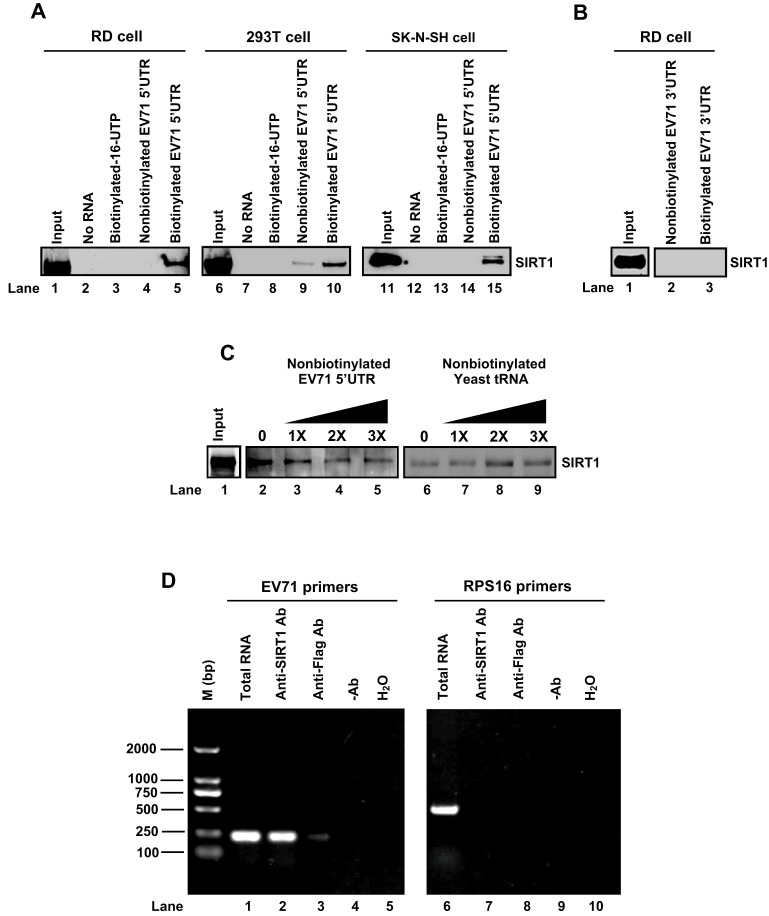
**SIRT1 binds directly to EV71 5′UTR, but not 3′UTR.** (A–C) Cell extracts of RD, 293T or SK-N-SH cells were prepared and used as inputs, or incubated with no RNA, biotin-16-UTP, non-biotinylated EV71 5′UTR or biotinylated EV71 5′UTR (A). RD cell lysates were prepared and used as input, or were incubated with nonbiotinylated EV71 3′UTR RNA or biotinylated EV71 3′UTR RNA (B). RD cell lysates were prepared and used as input, or were incubated with biotinylated EV71 3′UTR RNA along with different concentrations of non-biotinylated EV71 3′UTR RNA or nonbiotinylated yeast tRNA (C). Protein–RNA pulldown assays were carried out with anti-SIRT1 antibody and precipitated with protein G. Interactions between SIRT1 and EV71 5′UTR were determined by western blotting with anti-SIRT1 antibody. (D) RD cells were infected with EV71 at an MOI of 10 for 12 h. Cell extracts were prepared and used for mRNA RNA extraction (Total RNA), or were used for protein–RNA pulldown assays with anti-SIRT1 antibody, anti-Flag antibody, without antibody or with water and followed by mRNA extraction. Then standard RT-PCR analysis using primers specific to EV71 5′UTR RNA or ribosomal protein S16 (RPS16) was performed.

EV71 5′UTR contains a cloverleaf structure (stem-loop I) that is involved in viral RNA transcription and an IRES (stem-loops II–VI) that is required for translation initiation (Rohll et al., 1994). As the SIRT1 catalytic domain possesses a larger NAD^+^-binding subdomain with a Rossmann fold and a smaller helical module subdomain with a Zn^2+^-binding module (Min et al., 2001), we speculated that SIRT1 interacts with the EV71 5′UTR through this structure, leading to regulation of viral RNA transcription and translation. The ability of SIRT1 to bind to EV71 5′UTR was determined by RNA–protein pulldown assays. SIRT1 was detected in inputs (lysates of 293T, RD and SK-N-SH cells) (Fig. 6A, lanes 1, 6 and 11) and upon pulldown with biotinylated EV71 5′UTR (Fig. 6A, lanes 5, 10 and 15), but not in the absence of RNA (Fig. 6A, lanes 2, 7 and 12) or upon pulldown with biotin-16-UTP (Fig. 6A, lanes 3, 8 and 13) or non-biotinylated EV71 5′UTR (Fig. 6A, lanes 4, 9 and 14), suggesting that SIRT1 interacts with the EV71 5′UTR. EV71 RNA also contains a 3′UTR and a poly(A) tail. Given that EV71 3D^pol^ primes initiation of RNA replication at 3′ termini [3′UTR and poly(A) tail] of viral RNA, we determined whether the 3′UTR and poly(A) tail were involved in regulating EV71 replication. SIRT1 was detected in the input (Fig. 6B, lane 1), but not in the pulldown with non-biotinylated EV71 3′UTR (Fig. 6B, lane 2) or biotinylated EV71 3′UTR (Fig. 6B, lane 3), indicating that SIRT1 cannot interact with EV71 3′UTR.

To confirm the interaction between SIRT1 and EV71 5′UTR, we used an RNA–protein pulldown competition assay with non-biotinylated EV71 5′ UTR (as a specific competing probe) or yeast tRNA (as a nonspecific competing probe). SIRT1 was detected in input (Fig. 6C, lane 1), and its level was decreased upon pulldown in experiments with increasing amounts of non-biotinylated EV71 5′UTR (Fig. 6C, lanes 2–5), but remained relatively unchanged with increasing amounts of yeast tRNA (Fig. 6C, lanes 6–9), indicating that SIRT1 directly and specifically binds to the EV71 5′UTR. The ability of SIRT1 to bind to the EV71 5′UTR was further examined by co-immunoprecipitation and RNA–protein pulldown assays in EV71-infected RD cells. EV71 5′UTR (Fig. 6D, lane 1) and RPS16 RNA (Fig. 6D, lane 6) were detected in the input. The EV71 5′UTR (Fig. 6D, lane 2), but not RPS16 RNA (Fig. 6D, lane 7), was detected upon co-immunoprecipitation with anti-SIRT1 antibody. EV71 5′UTR and RPS16 RNA (Fig. 6D, lanes 3–5 and lanes 8–10) were not detected upon co-immunoprecipitation with anti-Flag antibody or without antibody. Taken together, these results show that SIRT1 binds directly to EV71 5′UTR RNA both *in vitro* and *in vivo*.

### SIRT1 represses EV71 IRES by binding to stem-loop I, II and III of EV71 5′UTR

**Fig. 7.**
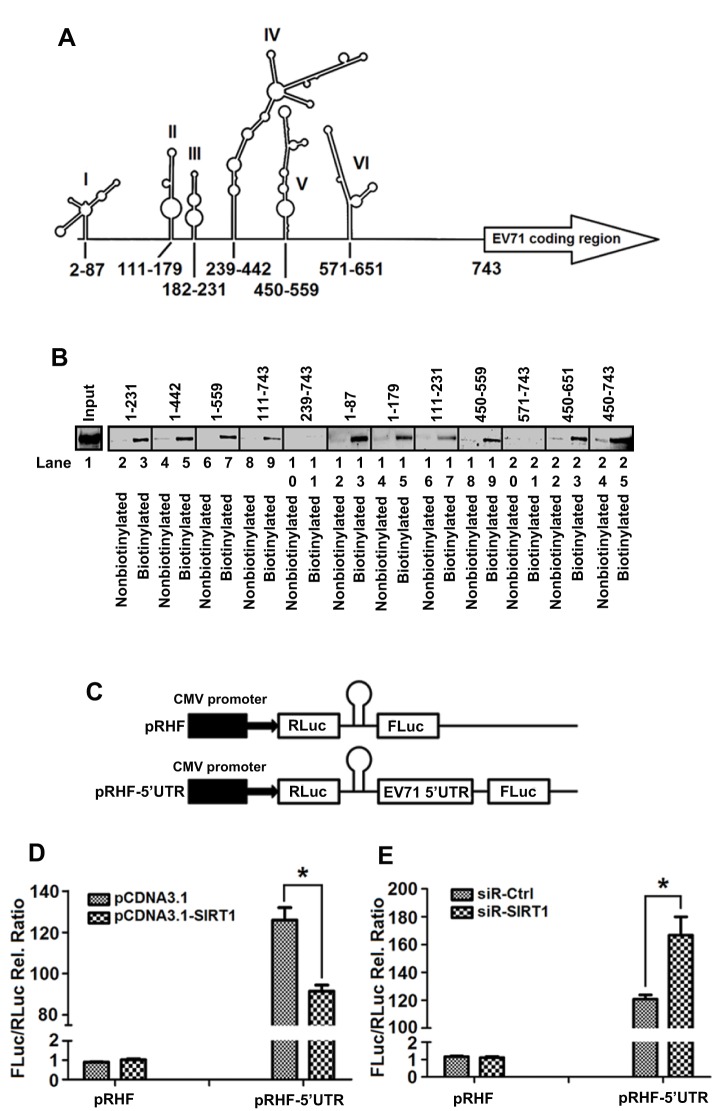
**SIRT1 represses EV71 IRES activity by binding to stem-loop I, II and III of the 5′UTR.** (A) Schematic diagram of EV71 5′UTR secondary structure predicted by M-FOLD software. Numbers indicate the first and last nucleotides in each stem-loop. (B) RD cell lysates were prepared and used as input. Cell lysates were incubated with various truncated constructs of a non-biotinylated EV71 5′UTR or biotinylated EV71 5′UTR. Mixtures were pulled down by streptavidin beads, and SIRT1 in RNA–protein complex was detected by western blotting with anti-SIRT1 antibody. (C) Schematic diagram of the two dicistronic reporter plasmids pRHF and pRHF-5′UTR. pRHF expresses dicistronic mRNA comprising the *Renilla* luciferase (RLuc) gene at the first cistron and the Firefly luciferase (FLuc) gene at the second cistron (cytomegalovirus, CMV). In pRHF-5′UTR, translation of first cistronic gene (RLuc) is cap-dependent, whereas translation of second cistronic gene (FLuc) depends on IRES activity. A hairpin inserted downstream of first cistron prevents ribosome read-through. (D,E) Cells were co-transfected with pcDNA3.1(+)-SIRT1 and pRHF-5′UTR for 12 h (D) or co-transfected with siR-Ctrl or siR-SIRT1 and pRHF or pRHF-5′UTR for 12 h (E). Cells were lysed, and FLuc and RLuc activities were measured. Calculating the ratio of FLuc activity to RLuc activity yields relative IRES activity. Results are mean±s.e.m. (*n*=5). *P<0.05 (*t*-test).

To verify the role of EV71 5′UTR in interacting with SIRT1, we constructed a series of truncated 5′UTR constructs (Fig. 7A), which were labeled with biotin-16-UTP. An RNA–protein pulldown assay showed that SIRT1 interacted with the biotinylated 5′UTR constructs containing nucleotides 1–231, 1–442, 1–559, 111–743, 1–87, 1–179, 111–231, 450–559, 450–651 and 450–743 (Fig. 7B, lane 3, 5, 7, 9, 13, 15, 17, 19, 23 and 25), but not biotinylated 5′UTR 239–743 and 571–743 (Fig. 7B, lanes 11 and 21) or nonbiotinylated truncated 5′UTRs (Fig. 7B, even-numbered lanes), indicating that SIRT1 binds with EV71 5′UTR stem-loops I, II and III. In addition, loop V (450–559) binds to SIRT1, whereas loops IV, V and VI might act as a whole to form a special RNA secondary structure where loop V binds to SIRT1.

Given that IRES-mediated translation initiation maintains progression of EV71 RNA translation during viral infection, we evaluated the effect of SIRT1 on the regulation of EV71 IRES activity. A dicistronic reporter plasmid pRHF-5′UTR in which the translation of *Renilla* luciferase (RLuc) was cap-dependent whereas the translation of Firefly luciferase (FLuc) was dependent on EV71 IRES was used (Fig. 7C). RD cells were co-transfected with pRHF-5′UTR and pcDNA3.1(+)-SIRT1 or siR-SIRT1. IRES activity was significantly reduced (*P*<0.05) by SIRT1 (Fig. 7D), whereas it was significantly enhanced (*P*<0.05) by siR-SIRT1 (Fig. 7E), indicating that overexpression of SIRT1 downregulates IRES activity, and knockdown of SIRT1 upregulates IRES activity. Thus, SIRT1 represses EV71 IRES activity to inhibit viral RNA translation initiation by binding to its 5′UTR.

### SIRT1 is colocalized with EV71 RNA in cytoplasm

**Fig. 8.**
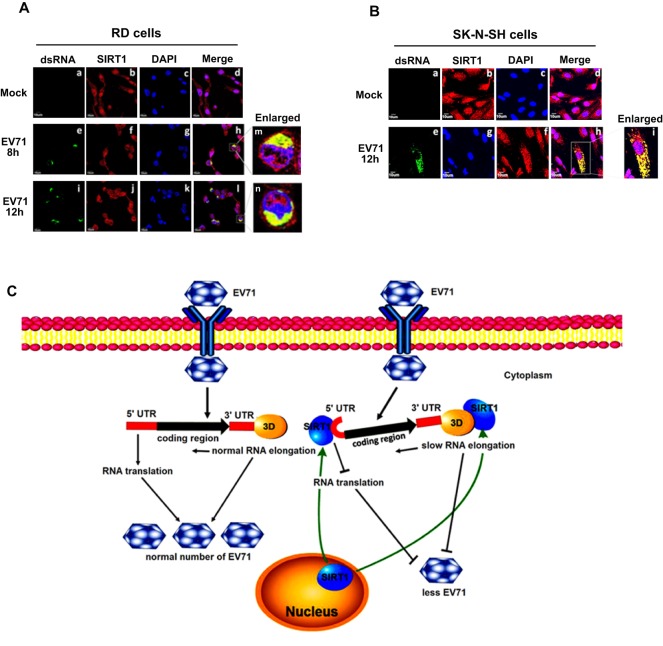
**SIRT1 is colocalized with EV71 RNA in cytoplasm.** (A) RD cells were infected or not (Mock) with EV71 at an MOI of 5 for 8 or 12 h. Cells were fixed, permeabilized and immunostained with antibody against EV71 dsRNA (a,e,i) or SIRT1 (b,f,j). FITC-conjugated donkey anti-mouse-IgG or Cy3-conjugated goat anti-rabbit-IgG was used as a secondary antibody. Nuclei were stained with DAPI (c,g,k). m and n show enlargements of h and l. Immunofluorescence was detected using a confocal laser-scanning microscopy. (B) SK-N-SH cells were infected with EV71 at an MOI of 20 for 12 h. Cells were fixed, permeabilized and immunostained with antibody against EV71 dsRNA (a,e) or SIRT1 (b,f). FITC-conjugated donkey anti-mouse-IgG or Cy3-conjugated goat anti-rabbit-IgG was used as a secondary antibody. Nuclei were stained with DAPI (c,g). i shows an enlargement of h. Immunofluorescence was detected with a confocal laser-scanning microscopy. (C) A proposed mechanism underlying the regulation of EV71 replication.

Because SIRT1 is mainly localized in the nucleus, whereas EV71 replication occurs in cytoplasm, we evaluated subcellular localization of SIRT1 during EV71 infection. RD and SK-N-SH cells (Fig. 8A,B) were infected with EV71, fixed, incubated with anti-EV71 dsRNA antibody or anti-SIRT1 antibody and examined under laser-scanning confocal microscopy. In mock-infected RD cells, EV71 dsRNA was not detected (Fig. 8Aa), and SIRT1 protein was mainly localized in the nucleus (Fig. 8Ab) but not in the cytoplasm (Fig. 8Ac,d). However, in RD cells infected with EV71 at 8 and 12 hpi, EV71 dsRNA was detected in cytoplasm (Fig. 8Ae,i), and a large proportion of the SIRT1 protein was colocalized with EV71 dsRNA in cytoplasm (Fig. 8Ah,l), but not in the nucleus (Fig. 8Af,j, enlarged in Fig. 8Am,n). Similarly, in mock-infected SK-N-SH cells, EV71 dsRNA was not detected (Fig. 8Ba) and SIRT1 was mainly localized in the nucleus (Fig. 8Bc,d). In EV71-infected SK-N-SH cells, EV71 dsRNA was detected in the cytoplasm (Fig. 8Be), and a large proportion of SIRT1 was colocalized with EV71 dsRNA in the cytoplasm (Fig. 8Bh), but not in the nucleus (Fig. 8Bf, enlarged in Fig. 8Bi). These results reveal that SIRT1 colocalized with EV71 RNA and translocated from the nucleus to the cytoplasm.

## DISCUSSION

Since EV71 was first described during an outbreak in California (Blomberg et al., 1974; Schmidt et al., 1974), its infection has emerged as a serious hazard that threatens the health of children and causes tremendous damage to both families and society. It is important to understand the mechanisms underlying viral infection and replication in order to prevent and control the disease. In this study, we investigated the mechanisms involved in the regulation of EV71 replication. We initially revealed that EV71 activates SIRT1 production and function, and further demonstrated that EV71 facilitates SIRT1 sumoylation to regulate its deacetylation activity. Although it has been reported that HPV E7 protein upregulates SIRT1 expression (Allison et al., 2009), HIV-1 Tat protein potently inhibits SIRT1-mediated deacetylation (Kwon et al., 2008) and HCV core protein influences the activity of SIRT1 (Yu et al., 2013), the role of SIRT1 in EV71 replication was unknown. We thus speculated that SIRT1 might play a role in EV71 replication. In the process of exploring such speculation, we demonstrated that SIRT1 plays an inhibitory role in EV71 replication.

It is known that SIRT1 is mainly localized in the nucleus, whereas EV71 replication occurs in the cytoplasm. Thus, we evaluated how SIRT1 regulates EV71 replication. Interestingly, in EV71-infected cells, SIRT1 changes its location from the nucleus to the cytoplasm. We speculated that such translocation might affect SIRT1 function in the regulation of EV71 replication. To confirm this speculation, we determined the functions of wild-type SIRT1 and mtNLS-SIRT1 in EV71-infected cells. Surprisingly, mtNLS-SIRT1 inhibited EV71 protein production and RNA replication. We further revealed that in EV71-infected cells, SIRT1 was colocalized with viral RNA and translocated from the nucleus to the cytoplasm. Therefore, we demonstrated that during EV71 infection, SIRT1 changes its location from the nucleus to the cytoplasm to bind to the viral RNA.

Moreover, the mechanism by which SIRT1 represses EV71 replication was evaluated. It was known that SIRT1 is an NAD^+^-dependent deacetylase (Landry et al., 2000), and we revealed that EV71 stimulates the SIRT1 sumoylation that is required for the deacetylase activity of SIRT1. Thus, we believed that SIRT1 might interact with EV71 proteins to regulate their functions. Our results demonstrated that SIRT1 specifically interacts with EV71 3D^pol^. The 3D^pol^ protein contains several domains, including the index finger, RING finger, pinky finger, middle finger, palm, and thumb domains (Wu et al., 2010). We further revealed that SIRT1 colocalized with 3D^pol^ in the cytoplasm through an interaction with its pinky finger domain. The pinky finger is the most conserved sub-domain among RdRps and forms the front side of the fingers and contains the G motif at the entrance of the template channel (Min et al., 2001). Thus, SIRT1 might play an important role in the regulation of 3D^pol^ RdRp activity.

The RdRp activity of EV71 3D^pol^ is essential for viral genome replication, including that of the negative-strand RNA and positive-strand RNA (Rueckert, 1996). The picornaviral 3D^pol^ is unique in the viral replication process and becomes active only upon complete proteolytic processing of the precursor 3CD^pro^ (Andino et al., 1993; Marcotte et al., 2007). Here, we evaluated the effects of SIRT1 on the function of EV71 3D^pol^. Because SIRT1 is a deacetylase, we determined its effects on the acetylation of 3D^pol^ using acetylation assays and deacetylation assays as described previously (Guo et al., 2012). Our results demonstrated that SIRT1 represses the acetylation of 3D^pol^. The effects of SIRT1-mediated deacetylation of 3D^pol^ on the RdRp activity was then revealed by an RNA elongation assay, as previously described (Chen et al., 2009; Hung et al., 2010), but modified to detect RNA synthesis by DIG-16-UTP labeling instead of isotope labeling (Xiao et al., 2002; Yi et al., 2003). Interestingly, the results confirmed that SIRT1 represses 3D^pol^ RdRp activity through inhibiting acetylation. More importantly, the activation of SIRT1 mediated by resveratrol downregulates EV71 protein production, and inhibition of SIRT1 mediated by EX527 and nicotinamide upregulates viral protein production. Thus, SIRT1 inhibits EV71 replication by attenuating 3D^pol^ acetylation and RdRp activity.

SIRT1 possesses a canonical sirtuin fold composed of a larger NAD^+^-binding subdomain and a smaller helical module subdomain (Min et al., 2001; Davenport et al., 2014). We speculated that this structure might provide opportunity for SIRT1 to interact with EV71 RNA, resulting in the regulation of viral replication. Our results confirmed that SIRT1 directly interacts with the EV71 5′UTR, but not its 3′UTR. The EV71 5′UTR contains a cloverleaf structure (stem-loop I) involved in RNA transcription and an IRES (stem-loops II–VI) mediating translation initiation (Rohll et al., 1994). We further revealed that SIRT1 binds with the cloverleaf, stem-loop II and stem-loop III of the EV71 5′UTR, which indicates that the cloverleaf and IRES are required for SIRT1 to regulate EV71 replication. The cloverleaf structure is essential for negative-strand synthesis (Barton et al., 2001; Lyons et al., 2001) that requires a membrane-associated replication complex of viral RNA template along with viral and host proteins (Luo et al., 2014; Lin et al., 2008, 2009a,b; Huang et al., 2011). Interactions between SIRT1 and 5′UTR RNA might play a role in the regulation of EV71 RNA synthesis. We previously reported that polyC-binding protein 1 (PCBP1) interacts with the cloverleaf of EV71 to facilitate viral RNA transcription (Song et al., 2015). We speculated that SIRT1 might compete with PCBP1 for the binding to EV71 5′UTR. This speculation was confirmed by the results showing that SIRT1 indeed competes with PCBP1 in binding to the cloverleaf of EV71 5′UTR (data not shown). During EV71 infection, IRES mediates the initiation of translation, which maintains the progression of viral RNA translation. As we showed that SIRT1 also binds to IRES, we further evaluated the effect of SIRT1 on IRES activity. Our results showed that SIRT1 represses the activity of the EV71 IRES. Therefore, SIRT1 inhibits EV71 RNA transcription by binding with EV71 5′UTR cloverleaf structure, and weakens viral RNA translation by interacting with EV71 5′UTR IRES.

In conclusion, we have revealed a new mechanism underlying the regulation of EV71 replication (Fig. 8C). During viral infection, EV71 stimulates SIRT1 production, sumoylation and a translocation from the nucleus to the cytoplasm, and attenuates the SIRT1 acetylase activity. Viral-activated SIRT1 subsequently interacts with EV71 3D^pol^ to repress 3D^pol^ acetylation and RdRp activity, leading to the attenuation of viral genome replication. SIRT1 also binds to the cloverleaf structure and IRES of EV71 5′UTR, resulting in the repression of viral RNA transcription and translation initiation.

## MATERIALS AND METHODS

### Cell lines and viruses

Human rhabdomyosarcoma (RD) cells and human neuroblastoma (SK-N-SH or A375) cells were purchased from the China Center for Type Culture Collection (CCTCC; Wuhan, China) and cultured in modified Eagle's medium (MEM). Human embryonic kidney (HEK-293T) cells were obtained from the American Type Culture Collection (ATCC; Manassas, VA) and cultured in Dulbecco's modified Eagle's medium (DMEM; Gibco BRL, Grand Island, NY) supplemented with 10% fetal calf serum (Gibco BRL), 100 U/ml penicillin and 100 µg/ml streptomycin sulfate. Cells were maintained at 37°C in a 5% CO_2_ incubator.

Enterovirus 71 Xiangyang strain (GenBank accession number JN230523.1) isolated by our group (Song et al., 2015) was used in this study. Virus infection was carried out as described previously. Briefly, cells were infected with EV71 at the indicated MOI after serum-starving overnight. Unbound virus was washed away for 1.5 h, and then cells were cultured with fresh medium supplemented with 2% fetal calf serum (FCS).

### Plasmids, small interfering RNAs, antibodies and reagents

The pcDNA3.0-5′UTR and the pcDNA3.0-3′UTR plasmids were amplified from EV71 cDNA with a PCR with the following primers: 5′UTR-F, 5′-ATTAAGGTTTTAAACAGCTGTGGGTTGTCA-3′ and 5′UTR-R, 5′-AATTCTAGAGGTTTTGCTGTGTTGAGGGT-3′; 3′UTR-F, 5′-GCAAGCTTTAGAGGCTATACACACCTCG-3′ and 3′UTR-R, 5′-GCTCTAGAGCTATTCTGGTTAT-3′. Then the DNA fragment was inserted into the *Hin*dIII and *Xba*I sites of the pcDNA3.0 vector.

The SIRT1-expressing plasmid pcDNA3.1-SIRT1 was kindly provided by Tony Kouzarides (University of Cambridge, Cambridge, UK). The reporter plasmids pRHF and pRHF-5′UTR were kindly provided by Shin-Ru Shih (Chang Gung University, Taiwan). The mtNLS-SIRT1 was mutated and used to construct a pcDNA3.1 vector with *Eco*RV and *Bam*HI sites. To construct plasmids expressing 2A, 2B, 2C, 3A, 3AB, 3C and 3D^pol^, fragments of EV71 cDNA were cloned into the *Hind*III and *Sal*I sites of a pEGFPC1 vector, resulting in GFP fusion proteins. Several plasmids for the expression and purification of recombinant proteins were also constructed. EV71 3D^pol^ was cloned into plasmid pGEX-6p-1 at the *Eco*RI and *Bam*HI restriction sites. SIRT1 and H363Y-SIRT1 were cloned into plasmid pET28a at the *Bam*HI and *Sal*I restriction sites, respectively. siSIRT1 was purchased from Ribobio.

### RNA extraction

Trizol was added to cells for 5 min at 37°C and samples were transferred to new centrifuge tubes. A one-fifth volume of chloroform was added, and samples were shaken and left to stand for 5 min at room temperature before being centrifuged at 12,000 ***g*** at 4°C for 15 min. Aqueous phases were transferred into new centrifuge tubes and isopycnic isopropanol was added, incubated for 5 min at room temperature, then centrifuged at 12,000 ***g*** at 4°C for 15 min. The supernatants were removed and the RNA pellets were washed in 1 ml 75% ethanol (750 µl absolute ethyl alcohol plus 250 µl DEPC H_2_O), then centrifuged at 12,000 ***g*** at 4°C for 5 min. The pellets were then air-dried and resuspended in DEPC-treated H_2_O and stored at −80°C until further use.

### Western blotting

Whole-cell extracts (30–120 µg) were separated by 8–12% SDS-PAGE. Protein concentration was determined using a Bradford assay (Bio-Rad). After electrophoresis, proteins were transferred onto a nitrocellulose filter membrane (Millipore). The membranes were blocked for 1 h at room temperature in 5% skim milk and then probed with the indicated primary antibodies at an appropriate dilution (Table S1) overnight at 4°C. The membranes were then incubated with secondary antibodies. Proteins were detected using a Luminescent Image Analyzer (Fujifilm LAS-4000).

### Pulldown assay using streptavidin beads and biotinylated RNA probes

The reaction mixtures contained 300 μg cell extracts and 3 μg biotinylated EV71 5′UTR RNA probe. Final reaction mixture volumes were adjusted to 100 μl with RNA mobility shift buffer (5 mM HEPES, 40 mM KCl, 0.1 mM EDTA, 2 mM MgCl_2_, 2 mM dithiothreitol, 1 U RNasin and 0.25 mg/ml heparin). The mixtures were incubated for 15 min at 30°C, and 400 μl Streptavidin MagneSphere Paramagnetic Particles (Promega) were added for binding at room temperature for 10 min. The RNA–protein complexes were washed six times with RNA mobility shift buffer (5 mM HEPES, 40 mM KCl, 0.1 mM EDTA, 2 mM MgCl_2_, 2 mM dithiothreitol, 1 U RNasin). Then 32 µl 1×PBS and 8 µl 5×SDS-PAGE loading buffer were added to reaction mixtures. Samples were boiled for 5 min, subjected to 12% SDS-PAGE, and then transferred to nitrocellulose membranes. The membranes were blocked for 1 h at room temperature in 5% skim milk in 1×PBST buffer, washed three times with 1×PBST buffer, and incubated with the anti-SIRT1 antibody overnight at 4°C. The membranes were washed three times with 1×PBST (PBS with 0.04% Tween 20) buffer, treated with a 1:5000 dilution of horseradish peroxidase (HRP)-conjugated anti-rabbit antibody for 45 min at room temperature, and washed six times with 1×PBST buffer. The membranes were incubated with HRP substrate, and then we proceeded with HRP color development.

### Immunoprecipitation and qRT-PCR

RD cells were infected with EV71 at an MOI of 10 for 10 h and cell extracts were prepared. Cell extracts were pre-cleared on ice for 1 h with protein-G–agarose in RIPA lysis buffer. The non-specific complexes were collected by centrifugation at 12,000 ***g*** at 4°C for 15 min. The supernatants were then collected for the immunoprecipitation assay. After protein concentration was measured using the Bradford assay (Bio-Rad), 300 μg pre-cleared lysate was diluted with 500 μl lysis buffer, 1 μg anti-SIRT1 or anti-Flag (as a negative control) antibody was added, and samples were incubated on ice for 2 h. Pre-washed protein-G–agarose was then added to each sample, which were then incubated on ice for 1 h. Immune complexes were pelleted by centrifugation at 12,000 ***g*** at 4°C for 5 min and washed three times with lysis buffer. Then 150 μg pre-cleared cell extract and other immune complexes were dissolved in Trizol, and RNA was extracted with chloroform and isopropanol. Quantitative real-time PCR (qRT-PCR) was performed with primers specific to the ribosomal protein S16 RNA or EV71 5′UTR RNA (RPS16, forward, 5′-GCGCGGTGAGGTTGTCTAGTC-3′ and reverse, 5′-GAGTTTTGAGTCACGATGGGC-3′; 5′UTR, forward, 5′-ACAATTAAAGAGTTGTTACCATATAGCTATTGGATTGGCC-3′ and, reverse, 5′-CATGTTTTGCTGTGTTGAGGGTCAAGAT-3′) or SIRT1, VP1 and β-actin (SIRT1, forward, 5′-GTTCAGCAACATCTTATGATTGGCA-3′ and reverse, 5′-TCAGGTATTCCACATGAAACAGACAC-3′; VP1, forward, 5′-AATTGAGTTCCATAGGTG-3′ and reverse, 5′-CTGTGCGAATTAAGGACAG-3′; and β-actin, foward, 5′-TGAAGTGTGACGTGGACATCCG-3′ and reverse, 5′-GCTGTCACCTTCACCGTTCCAG-3′).

### Co-immunoprecipitation and GST pulldown

Transfected cells were pelleted by centrifugation at 3000 ***g*** for 5 min, then re-suspended and sonicated in 1 ml RIPA lysis buffer. Lysates were centrifuged at 20,000 ***g*** for 15 min to remove cellular debris. Antibody and protein-G–agarose were added to supernatants, which were then gently shaken overnight at 4°C. The protein-G–agarose was then washed with RIPA wash buffer six times, and 32 µl 1×PBS and 8 µl 5×SDS-PAGE loading buffer were added prior to the SDS-PAGE and western blot procedures.

For the GST pulldown assay, 2 µg purified recombinant GST–3D^pol^ protein or purified GST protein was incubated with 2 µg purified recombinant His–SIRT1 protein and 40 µl glutathione resin (GenScript) in binding buffer (20 mM Tris-HCl pH 8.0, 500 mM NaCl, 1 mM DTT, 0.1 mM EDTA, 1 mM PMSF) overnight at 4°C. The mixtures were washed three times with wash buffer (20 mM Tris-HCl pH 8.0, 500 mM NaCl, 1 mM DTT, 0.1 mM EDTA), and 32 µl 1×PBS and 8 µl 5×SDS-PAGE loading buffer were added, as well as glutathione resin. The proteins were separated by SDS-PAGE and detected by performing western blot analysis.

### *In vitro* transcription

The T7-promoter EV71 5′UTR DNA fragment cleaved by the *Xba*I enzyme was excised from the vector pcDNA3.0-5′UTR. Transcription was performed using the T7 Quick High Yield RNA Synthesis Kit (NEB) according to the manufacturer's instructions. Biotinylated RNA was synthesized in 20 μl transcription reaction mixtures containing 0.5 μl 20 mM biotinylated UTP [biotin-16-UTP; Roche].

### Protein expression and purification

To construct pGEX6p-1-3D^pol^, the EV71 3D^pol^ region of EV71 was sub-cloned into the *Bam*H1 and *Eco*R1 restriction sites of the pGEX6p-1 vector. The plasmid was then transfected into *Escherichia coli* strain BL21 (DE3). Ampicillin-resistant colonies were grown in LB medium at 37°C until the OD_600_ reached 0.6–0.8. Isopropyl β-D-1-thiogalactopyranoside (IPTG) was added to a final concentration of 0.1 mM, and the cultures were incubated for an additional 16 h at 16°C. Cells were harvested by centrifugation, re-suspended, and sonicated in lysis buffer (20 mM Tris-HCl pH 8.0, 500 mM NaCl, 1 mM DTT, 0.1 mM EDTA and 1 mM PMSF). Lysates were centrifuged at 20,000 ***g*** for 30 min to remove cellular debris. Supernatants were loaded onto glutathione–Sepharose columns (GenScript) and gently shaken overnight at 4°C. After being washed with wash buffer (20 mM Tris-HCl pH 8.0, 500 mM NaCl, 1 mM DTT), recombinant GST–3D^pol^ protein was eluted using elution buffer (50 mM Tris-HCl pH 8.0, 150 mM NaCl, 5 mM EDTA, 0.1% Triton X-100, 9 mg/ml reduced glutathione, and 200 μg/ml PMSF) and stored at −80°C until further use.

The SIRT1 and H363Y-SIRT1 genes were cloned into the *Bam*HI and *Sal*I restriction sites of the vector pET28a encoding 6×His tag, to generate pET28a-SIRT1 and pET28a-H363Y-SIRT1, respectively. pET28a-SIRT1 and pET28a-H363Y-SIRT1 were transfected into BL21 (DE3) cells. The recombinant His–SIRT1 and His–H363Y-SIRT1 proteins were then induced with 0.1 mM IPTG for 6–8 h at 37°C. Cells were harvested and sonicated in 50 mM Tris-HCl pH 8.0, 250 mM NaCl, 1 mM β-mercaptoethanol and 200 μg/ml PMSF. Then His–SIRT1 and His–H363Y-SIRT1 proteins were loaded onto nickel nitrilotriacetic acid resin (Cwbiotech, cat. no. cw0010A), washed with wash buffer (50 mM Tris-HCl pH 8.0, 250 mM NaCl, 1 mM β-mercaptoethanol and 20 mM imidazole), eluted with 150 mM imidazole in elution buffer (50 mM Tris-HCl pH 8.0, 250 mM NaCl and 1 mM β-mercaptoethanol) and stored at −80°C until further use.

### 3D^pol^ activity and RNA elongation assay

The activity of EV71 3D^pol^ was determined using an elongation assay. The GST–3D^pol^ protein was incubated with RNA elongation assay buffer (50 mM HEPES, 5 mM DTT, 12.5 mM KCl, 5 mM MgCl_2_, 2 U/µl RNasin, 18 pmol oligo-dT18, 15 pmol polyA, 1 mM UTP, and 25 pmol DIG-UTP) for 30 min at room temperature. The reactions were terminated by adding EDTA to a concentration of 83 mM. DIG-labeled RNA was then spotted onto Hybond-N membrane and baked at 80°C for 2 h. The membrane was disposed as the DIG Northern Starter Kit provided by Roche and analyzed using the Luminescent Image Analyzer (Fujifilm LAS-4000).

### Immunofluorescence assay

RD cells were cultured in confocal dishes and infected with EV71 at an MOI of 5 for 12 h. Cells were fixed in a 1:1 mixture of methanol and acetone at 4°C for 20 min. After being washed three times with PBST, cells were incubated in 5% BSA in PBST at room temperature for 30 min and then incubated with anti-EV71 dsRNA antibody (1:100), anti-EV71 3D^pol^ antibody (1:100), or anti-SIRT1 antibody (1:100) for 3 h (see Table S1 for antibody details). Cells were washed in PBST three times and incubated with FITC-conjugated donkey anti-mouse IgG and Cy3-conjugated goat anti-rabbit-IgG (Proteintech Group) for 45 min at 37°C. Cells were washed three times in PBST, incubated with DAPI solution for 5 min at 37°C, and washed again with methanol three times and PBS three times. Finally, cells were analyzed using a confocal laser-scanning microscope (Olympus).

### Statistical analysis

All experiments were reproducible and carried out in duplicate or quadruplicate. Each set of experiments was repeated at least three times with similar results; representative experiments are shown. The results are presented as means. Student's *t*-test for paired samples was used to determine statistical significance. Differences were considered statistically significant at a *P*-value of ≤0.05.
